# Supplementary material for: A Critical View of the Application of the APEX Software (Aqueous Photochemistry of Environmentally-Occurring Xenobiotics) to Predict Photoreaction Kinetics in Surface Freshwaters
Source: Molecules. 2019 Dec 18;25(1):9. doi: 10.3390/molecules25010009 (PMC7017383; doi:10.3390/molecules25010009)
Supplement: Supplementary file 1 [file molecules-25-00009-s001.zip › 3.2.4_gcc-4.4.0.zip!/3.2.4_gcc-4.4.0/3.2.4_gcc-4.4.0/doc/octave/html/interpreter/Contributing-Guidelines.html]

Contributing Guidelines - Untitled


Next: Trouble,
Previous: Tips and Standards,
Up: Top


---

## Appendix D Contributing Guidelines

This chapter is dedicated to those who wish to contribute code to Octave.

- How to Contribute- General Guidelines- Octave Sources (m-files)- C++ Sources- Other Sources
